# Supplementary material for: Keto-anthraquinone covalent organic framework for H2O2 photosynthesis with oxygen and alkaline water
Source: Nat Commun. 2024 Mar 26;15:2649. doi: 10.1038/s41467-024-47023-y (PMC11258313; doi:10.1038/s41467-024-47023-y)
Supplement: Supplementary file 1 — Supplementary Information [file 41467_2024_47023_MOESM1_ESM.pdf]

Supplementary information

Keto-anthraquinone covalent organic framework for H_2O_2 photosynthesis with oxygen and alkaline water

Xiangcheng Zhang[1], Silian Cheng[1], Chao Chen[2], Xue Wen[1], Jie Miao[1], Baoxue Zhou[1], Mingce Long[1*], Lizhi Zhang[1*]

[1] School of Environmental Science and Engineering, Shanghai Jiao Tong University, Shanghai 200240, China

[2] School of Ecological and Environmental Science, Key Laboratory for Urban Ecological Processes and Eco-Restoration, East China Normal University, Shanghai 200241, China

*** Corresponding Author**

E-mail for Mingce Long: long_mc@sjtu.edu.cn

E-mail for Lizhi Zhang: zhanglizhi@sjtu.edu.cn

Supplementary Sections

Chemical materials

2,6-diaminoanthraquinone (AQ), 2,4,6-triformylphloroglucinol (Tp), pyromellitic aldehyde (LZU), 2,6-diaminoanthracene (DA) and acetonitrile were purchased from Shanghai Aladdin Bio-Chem Technology Co., Ltd. N, N-dimethylacetamide and 1,4-dioxane were obtained from Sigma-Aldrich and Sangon Biotech Co. Ltd., (Shanghai, China). N, N-dimethylformamide and acetone were purchased from Sinopharm Chemical Reagent Co. Ltd., (Beijing, China). Anhydrous MnO_2, Na_2SO_4, CH_3COONa and NaOH were obtained from Shanghai Titan Scientific Co. Ltd., (Shanghai, China). All the chemicals were of analytical grade and used as received without any further purification. Deionization water was used in all experiments.

Synthesis of TpDA

TpDA was synthesized via a solvothermal method. Typically, a 10 mL Schlenk tube was charged with 2,4,6-triformylphloroglucinol (Tp, 40 mg, 0.15 mmol) and 2,6-diaminoanthracene (DA, 62.4 mg, 0.15 mmol) in 1,4-dioxane (2 mL) with a catalytic amount of ice acetic acid (0.4 mL). The mixture was then sonicated for 10 min and degassed by freeze-pump-thaw three times. After the mixture was allowed to warm to room temperature, it was heated at 120 °C and left undisturbed for 72 h. The red precipitate was obtained by filtration and washed with N, N-dimethylformamide and acetone. Finally, the precursor COF was dried at 120 °C for 12 h in a vacuum oven. The obtained reference catalyst was denoted as TpDA.

Synthesis of LZUAQ

A 10 mL Schlenk tube was charged with pyromellitic aldehyde (LZU, 64 mg, 0.6 mmol) and 2,6-diaminoanthraquinone (AQ, 42.1 mg 0.30 mmol) in 1,4-dioxane (2 mL) with a catalytic amount of ice acetic acid (0.3 mL). The synthesis procedure was the same as that for TpDA. The obtained yellowish powder was denoted as LZUAQ.

Characterizations

Wide angle X-ray diffraction patterns (XRD) were conducted by a Rigaku D/max-

2200/PC diffractometer with a Cu-Kα radiation (λ=0.154 nm). Solid-state nuclear magnetic resonances (NMR) spectra of C^{13} were carried out on a Bruker BioSpin Avance III HD 400 high performance digital NMR spectrometer. X-ray photoelectron spectra (XPS) and Ultraviolet photoelectron spectroscopy (UPS) were implemented on an Axis Ultra DLD system (Shimadzu/Kratos) that was calibrated by C 1s at 284.6 eV. Raman spectra were recorded on a Bruker Senterra R200-L dispersive Raman microscope at 532 nm. Fourier transform infrared (FTIR) spectra were obtained from a Nicolet 6700 spectrometer (Thermo Electron). Transmission electron microscopy (TEM) images were obtained from a TECNA1 G2F20 microscope at an accelerating voltage of 200 kV. Scanning electron microscope (SEM) images were obtained on a Hitachi S-4800 microscope. The UV–vis diffuse reflectance spectrophotometer (DRS, Lambda 950, PerkinElmer, USA) was used to investigate the optical properties of catalysts. The steady-state and transient-state photoluminescence (PL) spectra were obtained on an Edinburgh-250 instrument with an excitation wavelength at 250 nm. Nitrogen adsorption–desorption isotherms were analyzed on a Micromeritics ASAP 2020 analyzer.

Kinetics analysis of H_2O_2

Kinetic analysis was carried out to assess the formation and decomposition rate constants of H_2O_2 during photocatalysis. The kinetic constants were calculated by fitting the H_2O_2 production curves via the following Box-Lucas model.

$$[H_2O_2] = (k_f/k_d)[1 - e^{(-k_d t)}] \qquad \text{(S1)}$$

$$y=A[1-e^{(-Bx)}]\ (k_d = B;\ k_f = A \times B) \qquad \text{(S2)}$$

Here, k_f is the zero-order H_2O_2 formation rate constant; k_d is the first-order H_2O_2 decomposition rate constant; t is the reaction time.

Apparent quantum yield (AQY)

The apparent quantum yield (AQY) was calculated by measuring H_2O_2 photosynthesis of Kf-AQ under different monochromatic light irradiation. The light intensities of 400, 450, 550, 600 and 650 nm were measured by an optical power meter (FZ-A, China). The AQY (%) was calculated by the equation S3.[1]

$$AQY\ (\%) = \frac{[H_2O_2 \text{ generated (mol)}]\times 2}{[\text{photon number entered into the reactor (mol)}]} \times 100\% \quad \text{(S3)}$$

The amount of H_2O_2 is presented in Table S3. The total photon number entering into the reactor can be calculated by the product of S, t, P, and λ, wherein, S is the irradiation area (m^2); t is the reaction time (3600 s); P is the incident monochromatic light intensity ($W\ m^{-2}$); λ is the wavelength of the incident monochromatic light (m), respectively.

Solar-to-chemical conversion (SCC) efficiency

For the investigation of SCC efficiency, 5 mg Kf-AQ was dispersed into 30 mL ultrapure water with different pH. After 1.0 h visible light (λ > 400 nm) illumination, the total incident power and amount of generated H_2O_2 were well calculated (Table S4). The SCC efficiency of Kf-AQ in different pH were determined by the equation S4,[2]

$$SCC(\%) = \frac{\triangle G_{H2O2} \times n_{H2O2}}{t_{ir} \times S_{ir} \times I_{cut420}} \times 100\% \quad \text{(S4)}$$

wherein, $\triangle G$ (117 kJ mol^{-1}) is represent the free energy of H_2O_2 generation; t_{ir} (3600 s) is represent the irradiation time; S_{ir} (3.74×10^{-3} m^2) is represent the irradiation area; I_{cut400} (984 W m^{-2}) is represent the incident light intensity, respectively.

Electron transfer number (*n*)

The electron transfer number for oxygen reduction reaction (ORR) was measured on a rotating disk electrode (RDE) in an O_2-saturated Na_2SO_4 (0.1 mol L^{-1}) system with different rotating speeds. The average numbers of electrons (n) were calculated by the Koutecky-Levich equation:[3]

$$\frac{1}{i} = \frac{1}{i_k} + [\frac{1}{0.620 nFAD^{2/3} v^{-1/6} C}]\omega^{-1/2} \quad \text{(S5)}$$

Here, i and i_k are the current density (μA cm^{-2}) and kinetic current density (μA cm^{-2}), respectively; n is the number of electron transfer; F is the Faraday constant (96485 C mol^{-1}); A is the working electrode area (0.196 cm^2); D is the oxygen diffusion coefficient (1.93×10^{-5} cm^2 s^{-1}); v is the kinematic viscosity of the electrolyte (0.0109 cm^2 s^{-1}); C is the saturated oxygen concentration in water (1.26×10^{-3} M); ω is the rotating speed.

Supplementary Figures

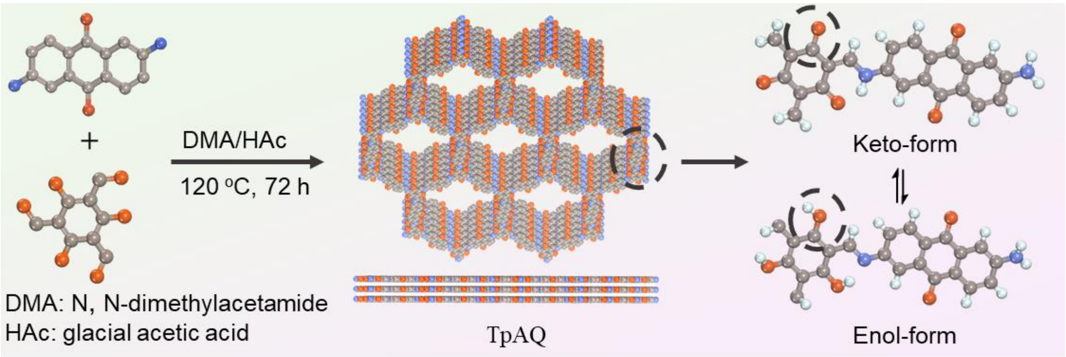

Figure S1. TpAQ synthesis. Scheme of TpAQ synthesis via solvothermal process.

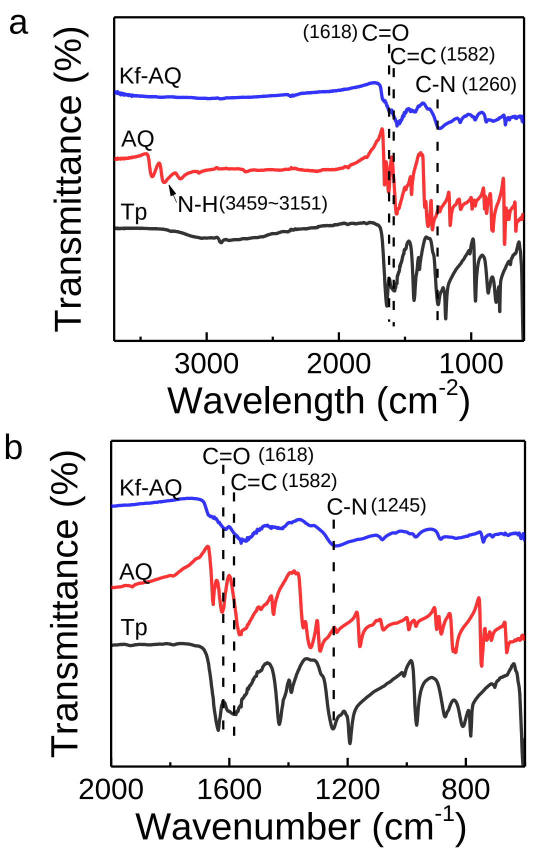

Figure S2. FTIR characterization. (a) FTIR spectra of Tp, AQ and Kf-AQ, (b) the amplified regions of FTIR spectra in the range from 600 cm^{-1} to 2000 cm^{-1}.

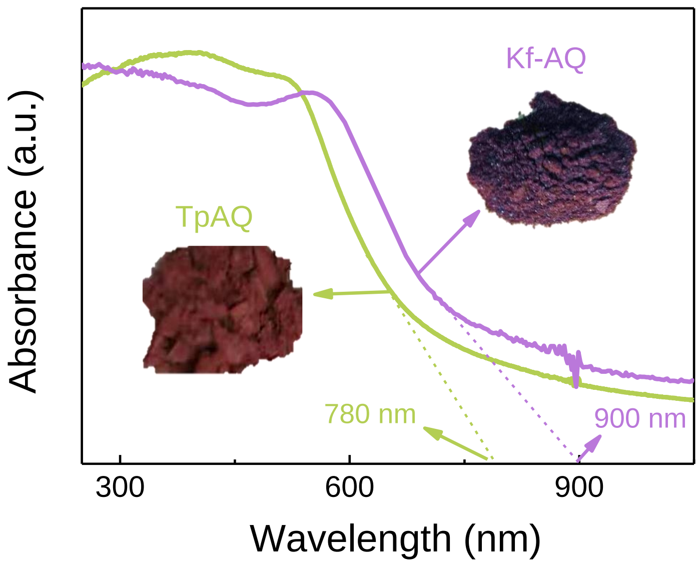

Figure S3. Light absorption property. The UV-visible diffuse reflectance spectrum (DRS) of Kf-AQ and TpAQ.

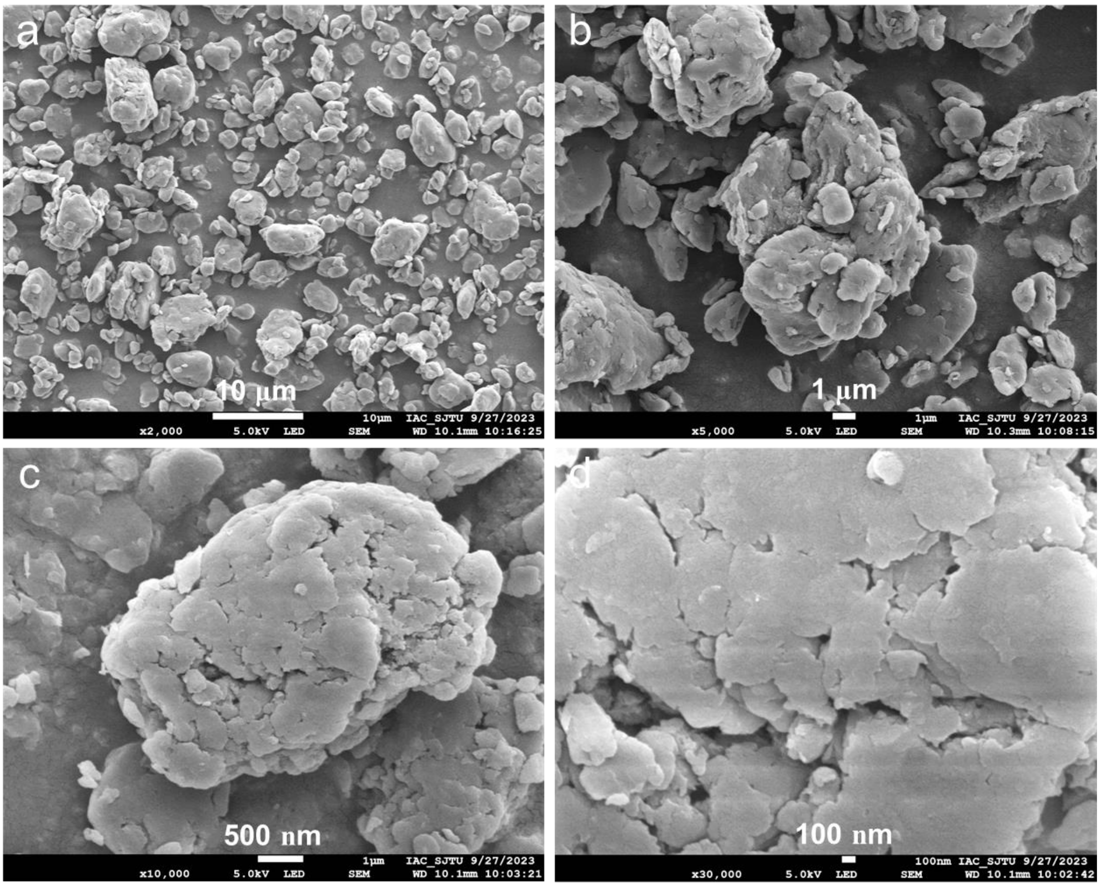

Figure S4. Microscopic morphology characterization. SEM images of Kf-AQ with different magnification: (a) ×2000; (b) ×5000; (c) ×10000; (d) ×30000.

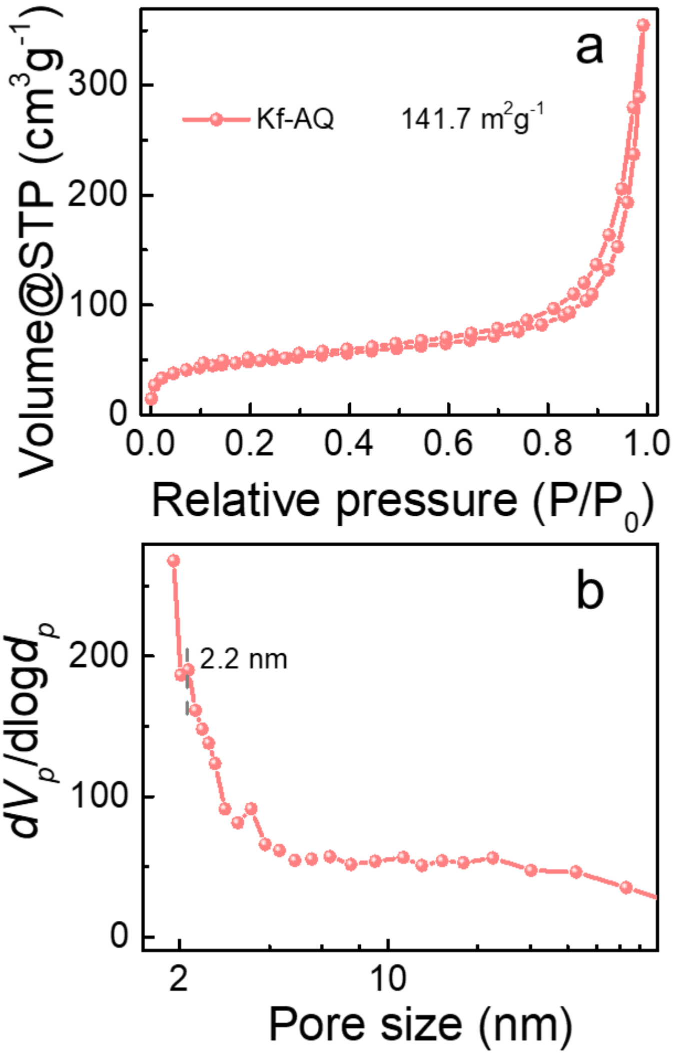

Figure S5. BET characterization of Kf-AQ. (a) N_2 adsorption-desorption isotherm and (b) pore size distribution of Kf-AQ. The specific surface area of Kf-AQ is calculated to be 141.7 m^2 g^{-1}, and the pore size is centered at 2.2 nm. The mesoporous distribution indicates the stacking structure of Kf-AQ.

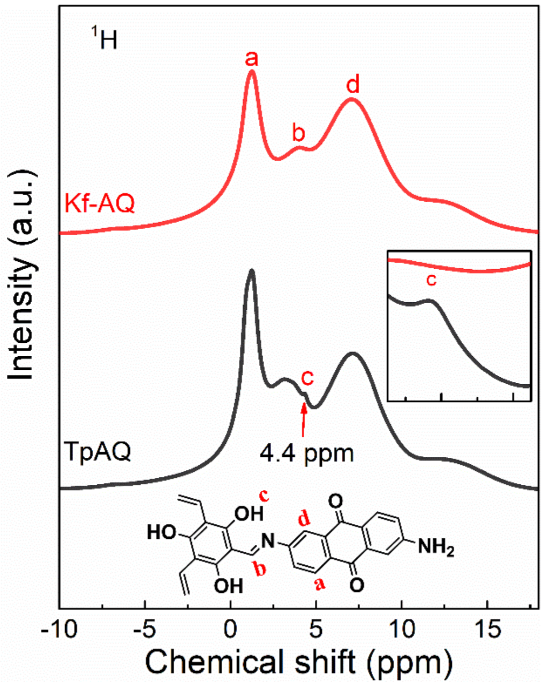

Figure S6. Solid-state ^{1}H NMR spectra. Solid-state ^{1}H NMR spectra of Kf-AQ and TpAQ.

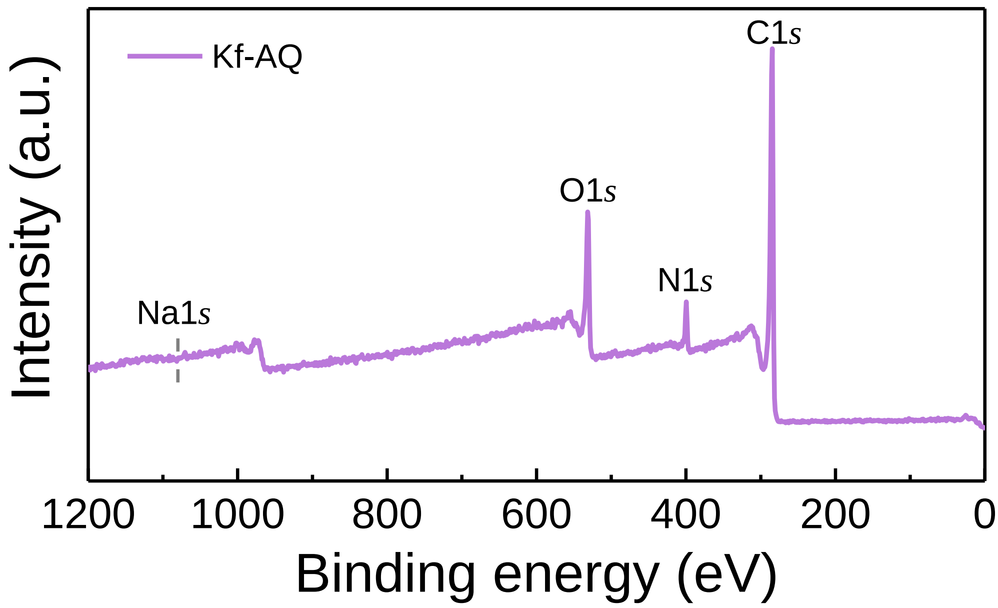

Figure S7. XPS survey spectrum. XPS survey spectrum of Kf-AQ.

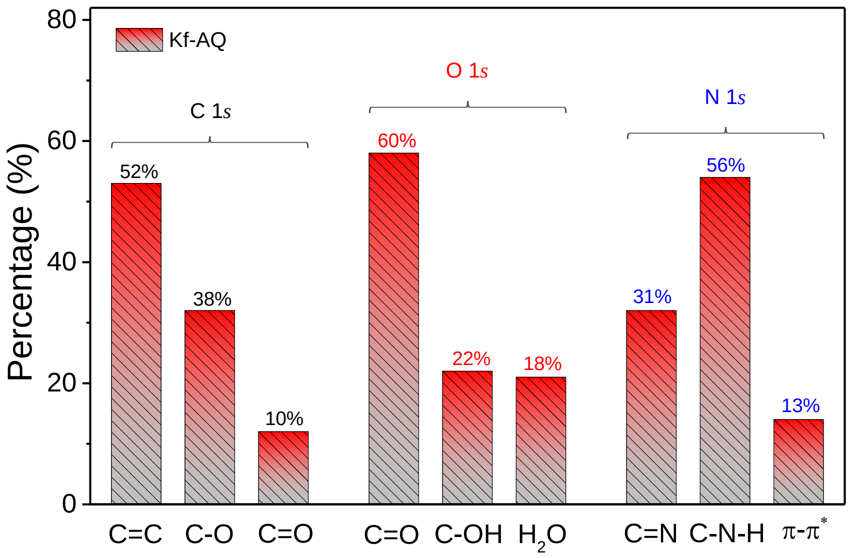

Figure S8. Analysis of different chemical bond proportions. The percentage of peak areas for different chemical bonds that were calculated from the deconvoluted C 1*s*, O 1*s* and N 1*s* XPS bands.

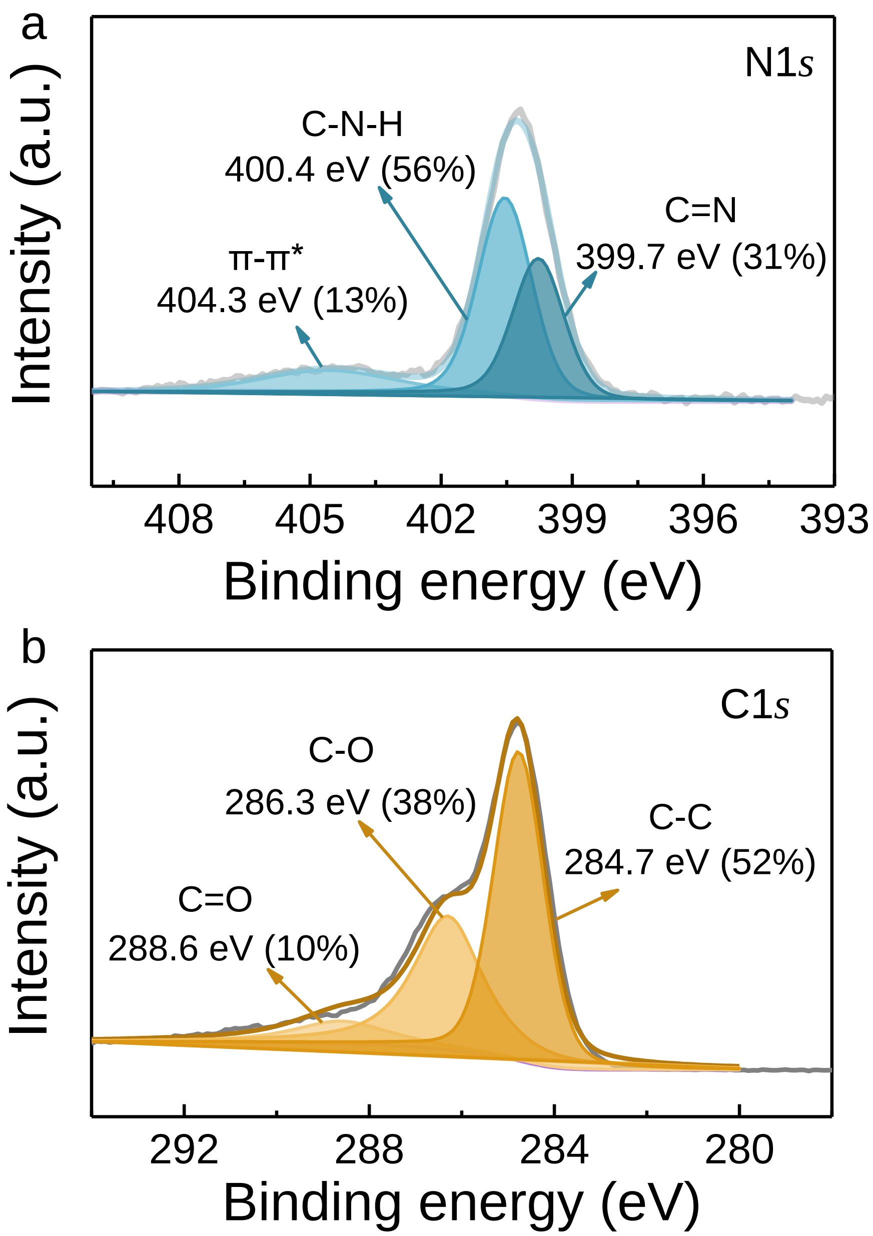

Figure S9. High-resolution XPS spectra. High-resolution N 1*s* (a) and C 1*s* (b) XPS spectra of Kf-AQ.

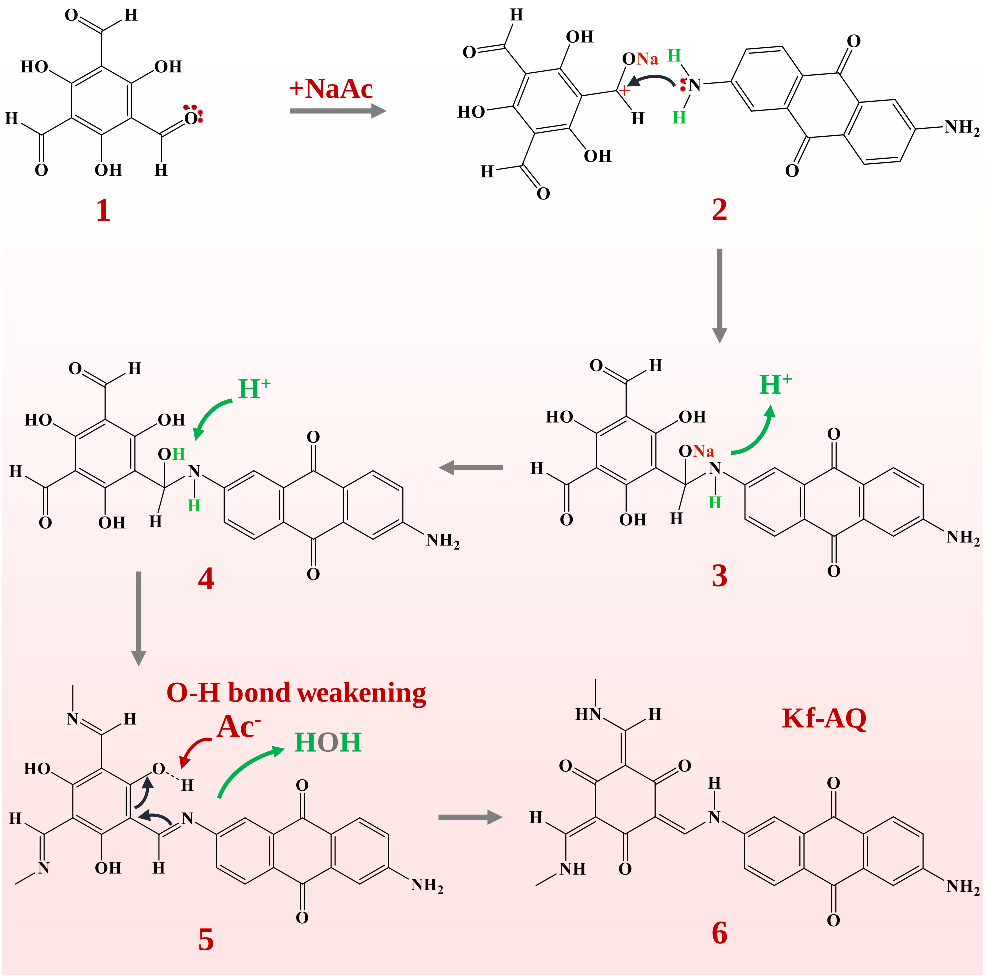

Figure S10. Synthesis mechanism. Probable synthetic mechanism of Kf-AQ.

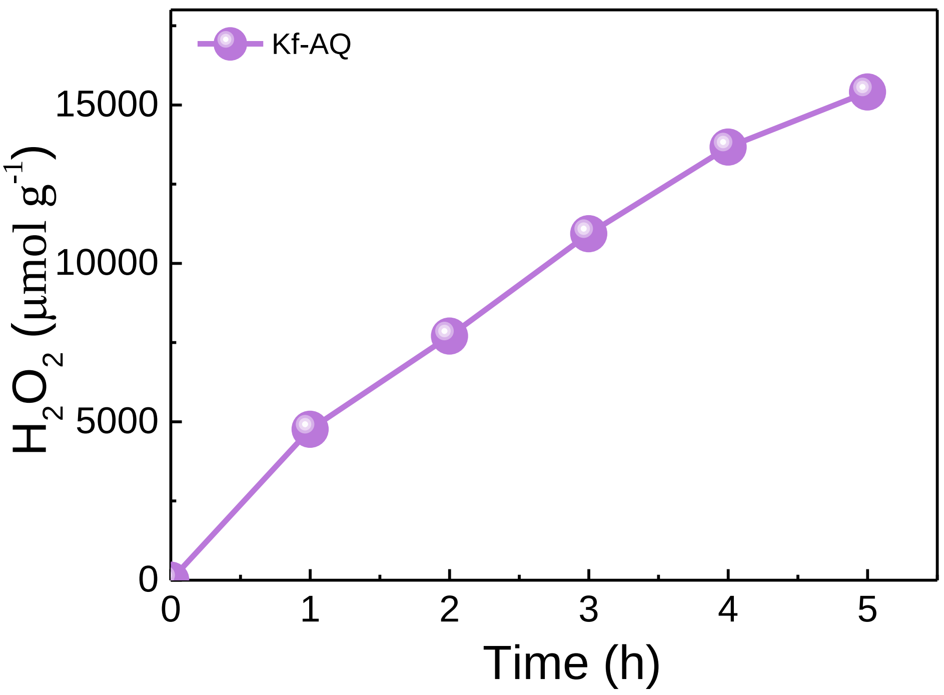

Figure S11. H_2O_2 photosynthesis. H_2O_2 photosynthesis of Kf-AQ in a prolonged test.

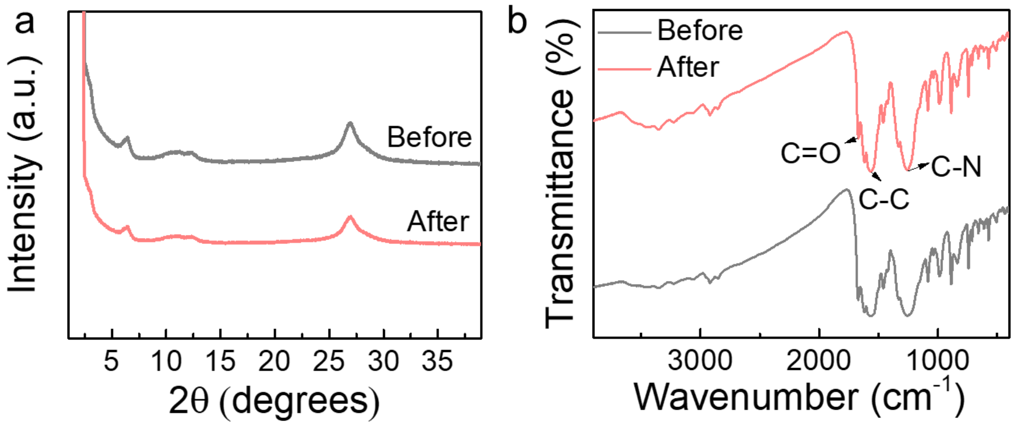

Figure S12. Stability analysis. The PXRD patterns (a) and FTIR spectra (b) of Kf-AQ before and after photoreaction.

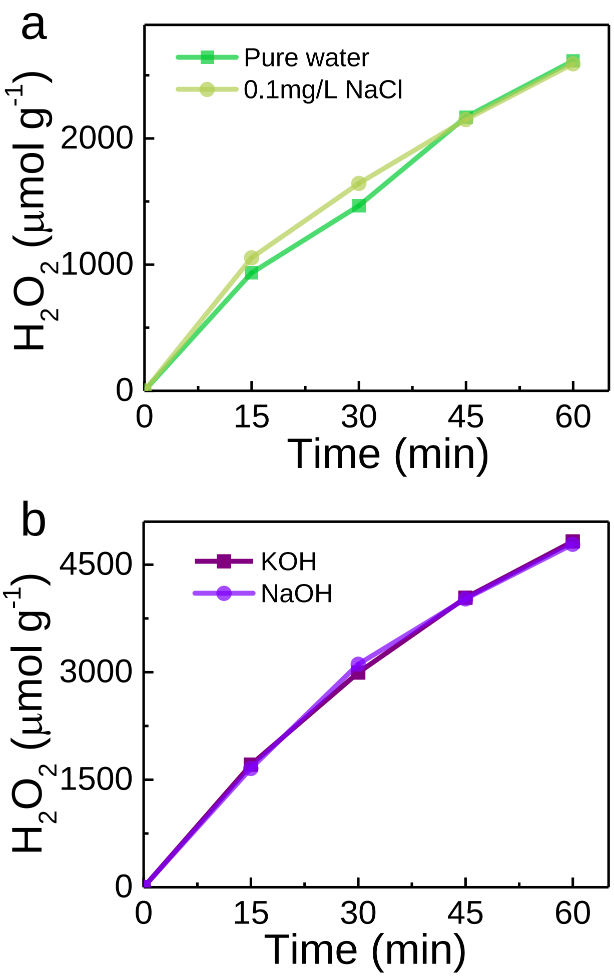

Figure S13. Effect of solution on H_2O_2 photosynthesis. H_2O_2 photosynthesis in various solution: (a) pure water and 0.1 mg L^{-1} NaCl; (b) KOH (pH=13) and NaOH (pH=13).

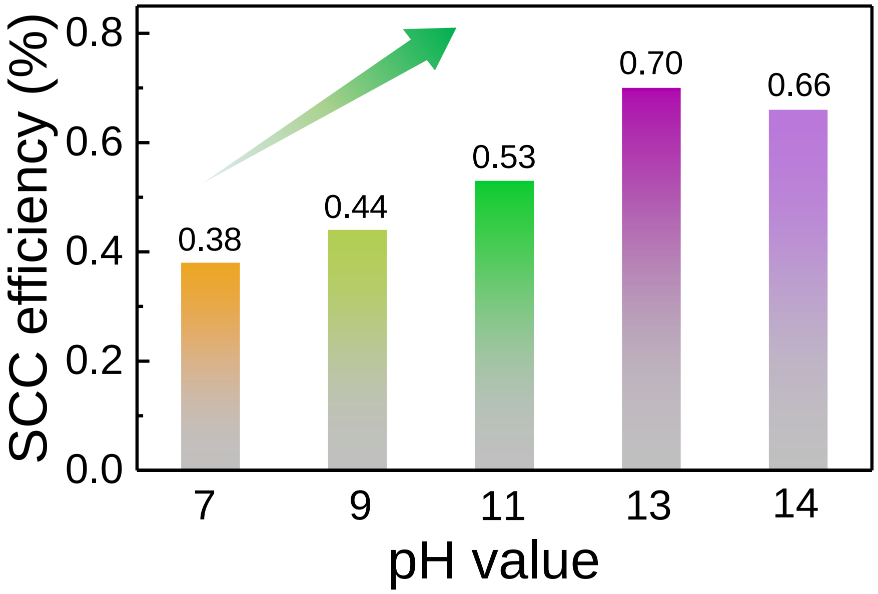

Figure S14. Efficiency analysis. Solar-to-chemical conversion (SCC) efficiencies of Kf-AQ under visible light ($\lambda > 400$ nm) illumination.

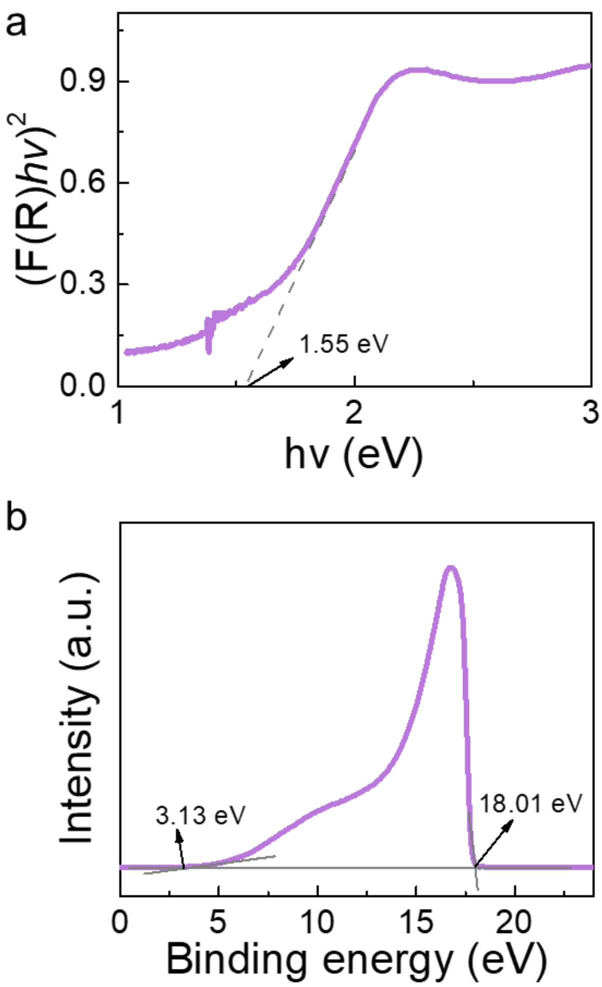

Figure S15. Energy band structure analysis. Tauc plot (a) and Ultraviolet photoelectron spectrum (b) of Kf-AQ.

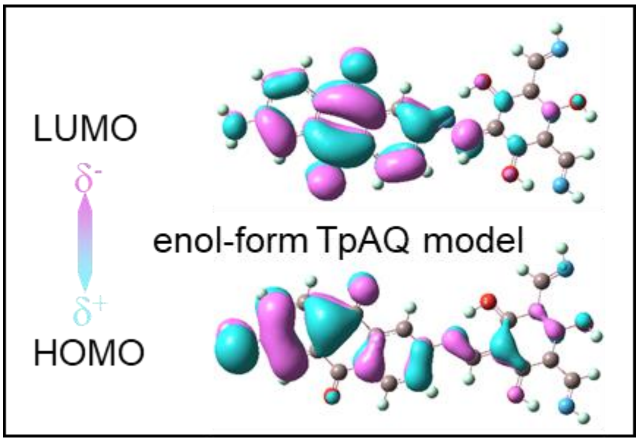

Figure S16. HOMO and LUMO calculation. Calculated HOMO and LUMO for enol-form TpAQ dimer.

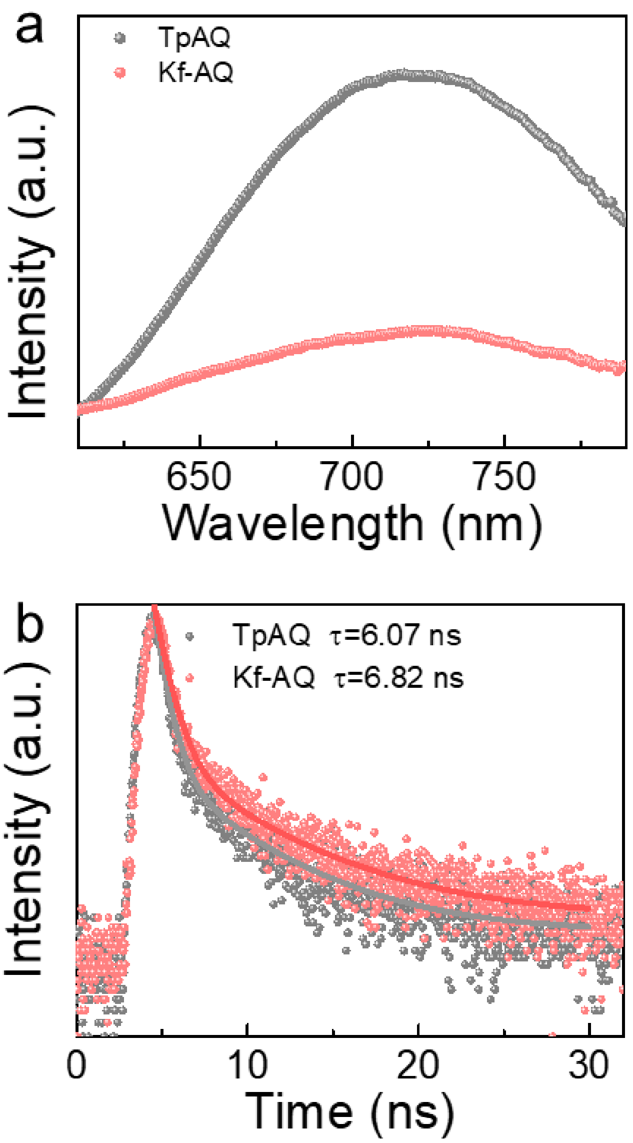

Figure S17. Analysis of photogenerated charge separation. The steady-state (a) and transient-state (b) fluorescence spectra of TpAQ and Kf-AQ.

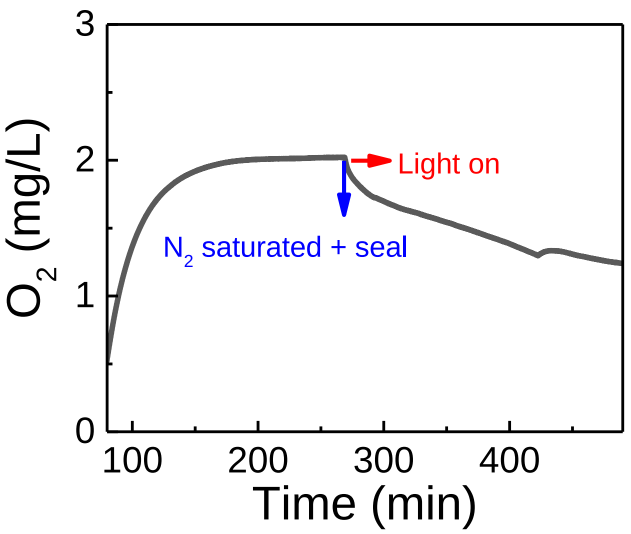

Figure S18. Variation of Oxygen concentration. Oxygen concentration in a Kf-AQ suspension at pH=13.

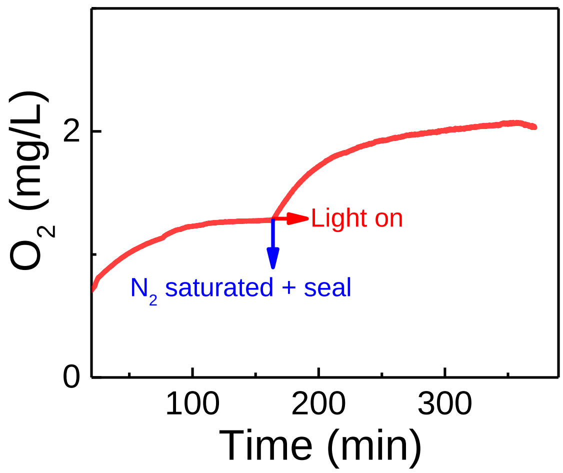

Figure S19. Variation of Oxygen concentration. Photocatalytic O_2 evolution in Kf-AQ suspension in the presence of $AgNO_3$ (0.16 g L^{-1}).

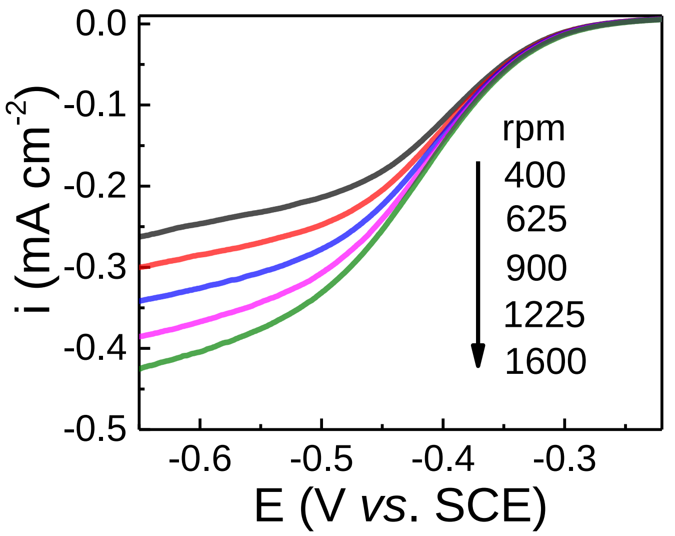

Figure S20. Electron transfer number analysis. LSV curves of Kf-AQ in an air saturated $NaClO_4$ (0.1 M) solution.

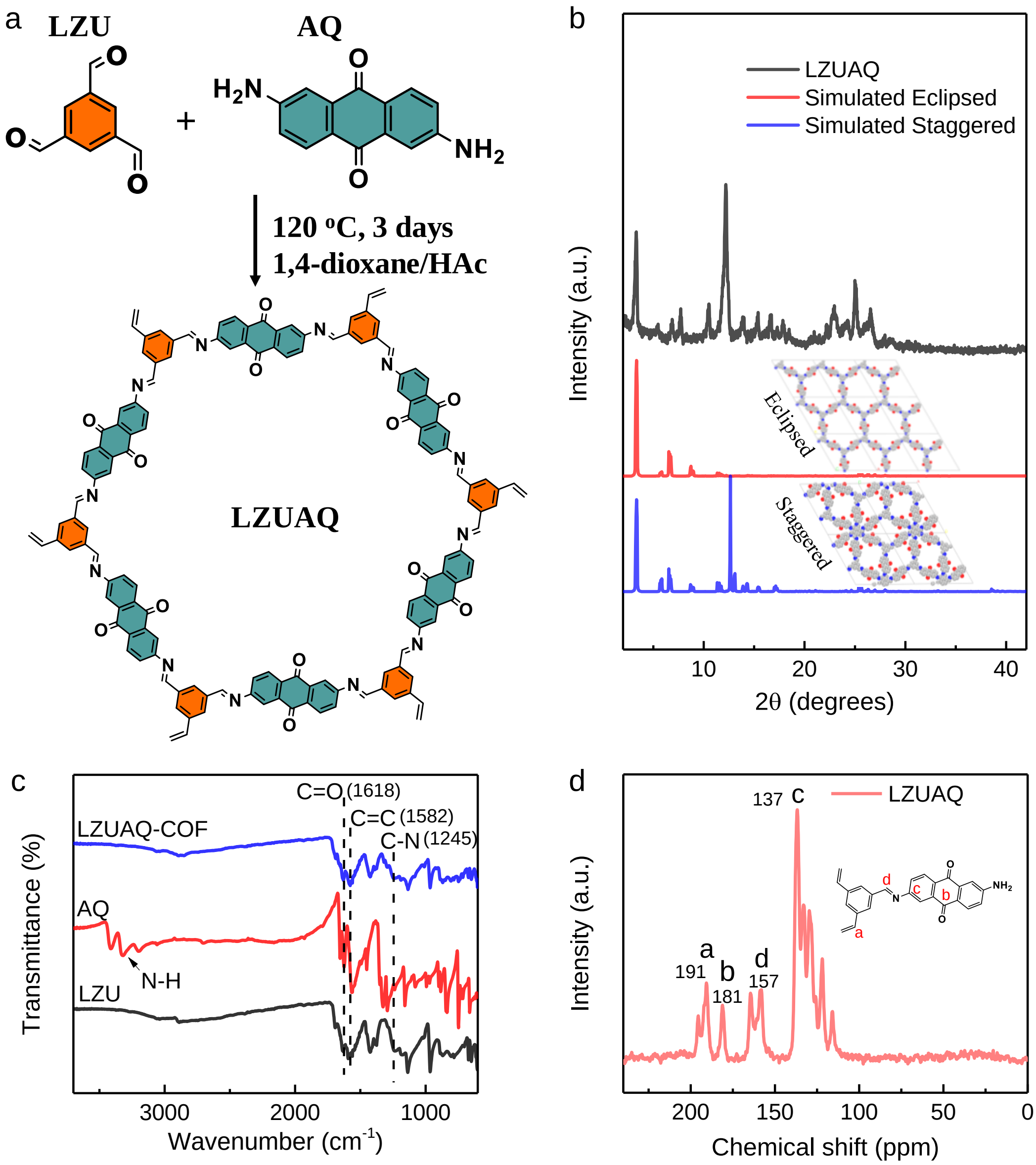

Figure S21. Synthesis process and structural characterization of LZUAQ. (a) Scheme of the LZUAQ condensation process. (b) PXRD patterns, experimentally observed (dark), simulated using eclipsed AA-stacking (red) and staggered AB stacking (blue) models. (c) FIRT spectra of LZU, AQ and LZUAQ, respectively. After condensation, the stretch peak of N-H in AQ disappeared, and the C=O at 1618 cm^{-1} in LZUAQ emerged, indicating the formation of conjugated structures. (d) ^{13}C NMR spectrum of LZUAQ. The chemical shift at 157 ppm was ascribed to C=N in the conjugated structure.

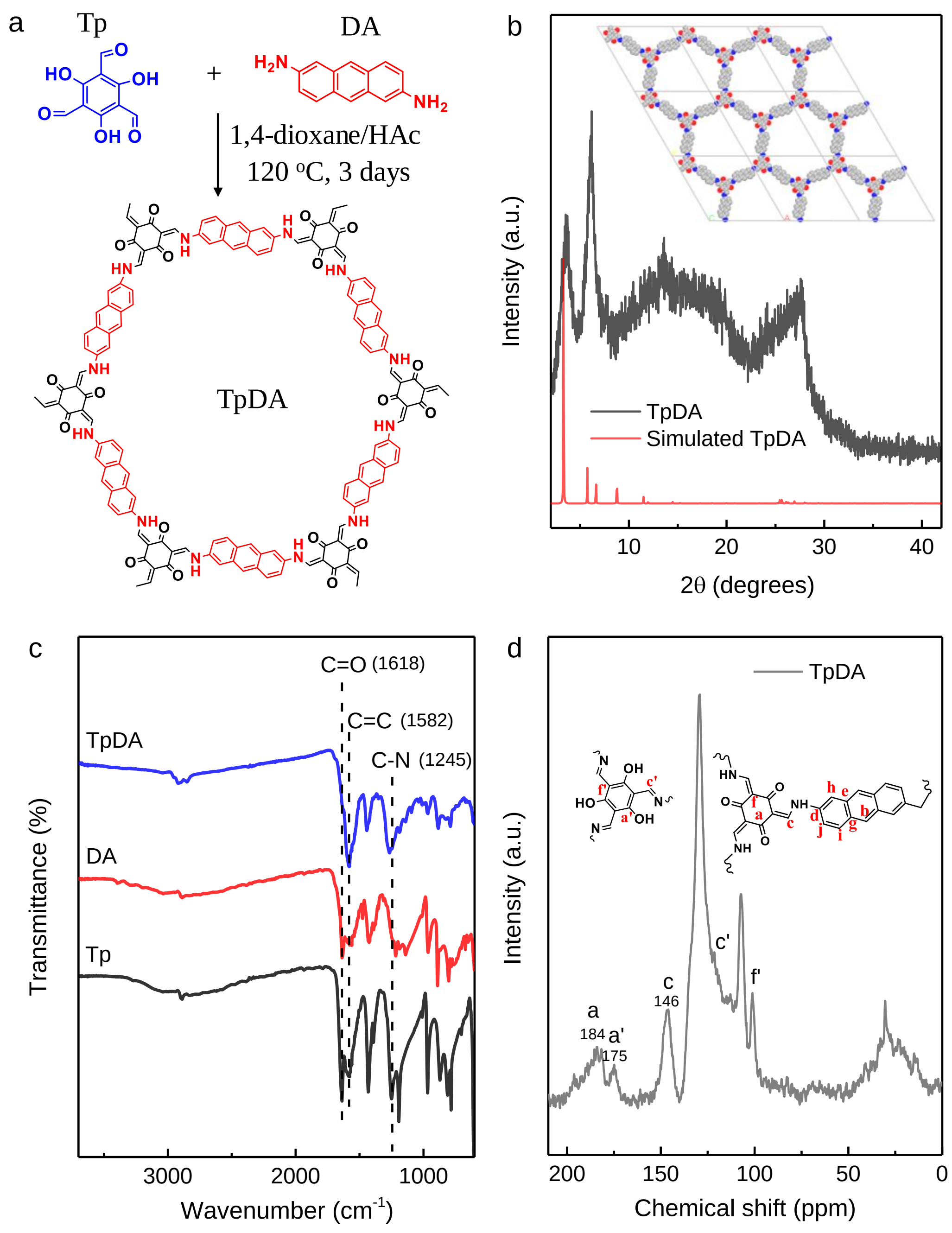

Figure S22. Synthesis process and structural characterization of TpDA. (a) Synthesis of the TpDA. (b) PXRD patterns, experimentally observed (dark), simulated using eclipsed AA-stacking (red) model. (c) FIRT spectra of Tp, DA and TpDA. FTIR spectra revealed the formation of a new C-N stretching band at 1245 cm^{-1}, ascribing to the Tp and DA conjugation. (d) ^{13}C NMR spectra of TpDA. The chemical shift at 184 ppm, 175 ppm and 146 ppm are ascribing to C=O, C-OH and C-NH-, respectively. The keto-form structure is dominated in the TpDA.

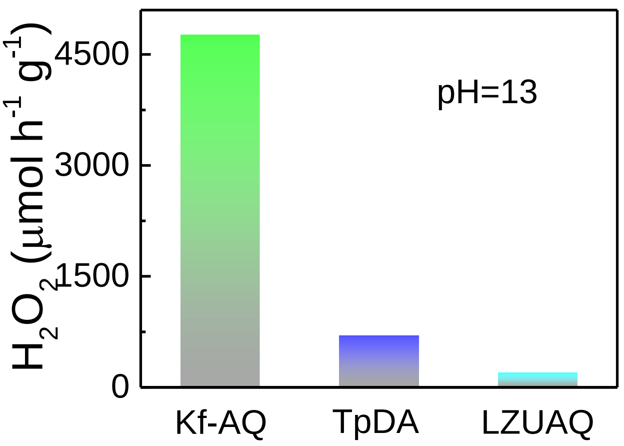

Figure S23. H_2O_2 photosynthesis. H_2O_2 photosynthesis of Kf-AQ, TpDA and LZUAQ.

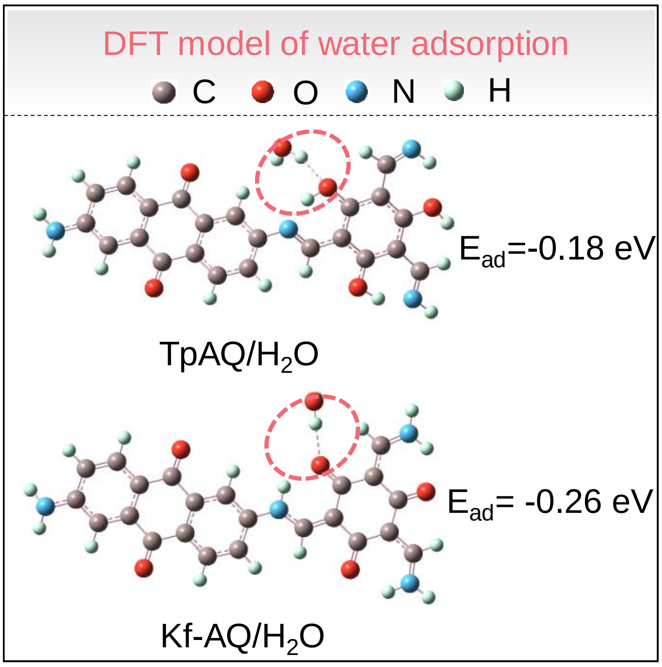

Figure S24. Adsorption energy calculation. The adsorption energy of one H_2O molecule over TpAQ and Kf-AQ.

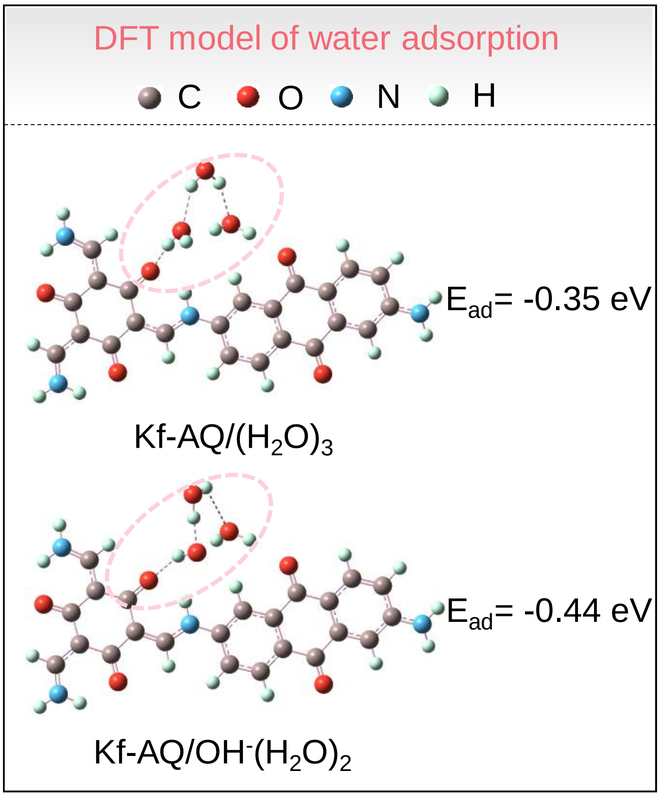

Figure S25. Adsorption energy calculation. The adsorption energy of different water clusters over Kf-AQ.

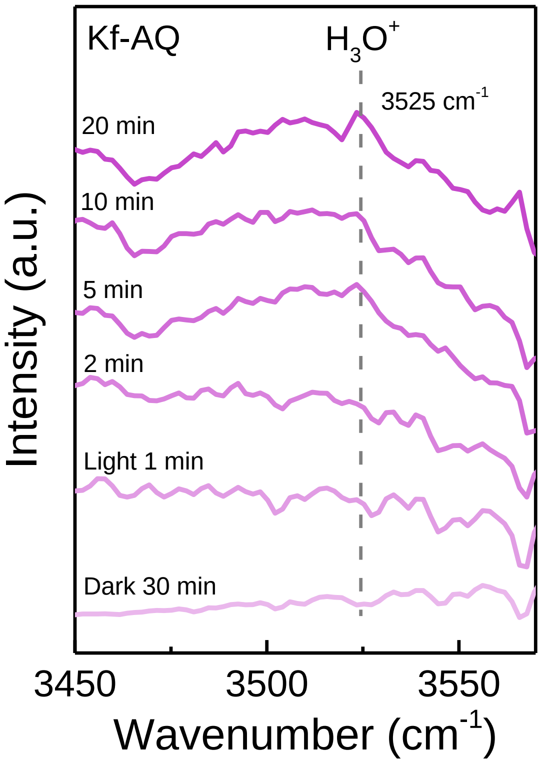

Figure S26. Hydrated hydrogen ion analysis. In-situ FTIR spectra of Kf-AQ under alkaline condition.

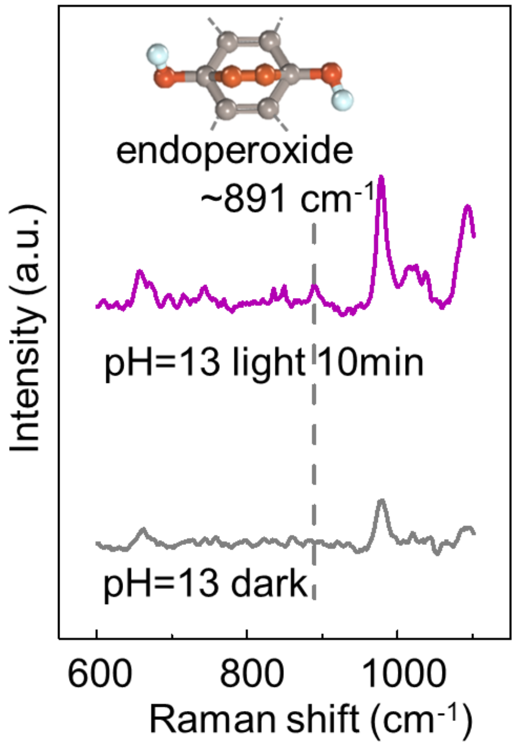

Figure S27. Endoperoxide analysis. Raman spectra of a Kf-AQ suspension under dark and intermittent visible light irradiation.

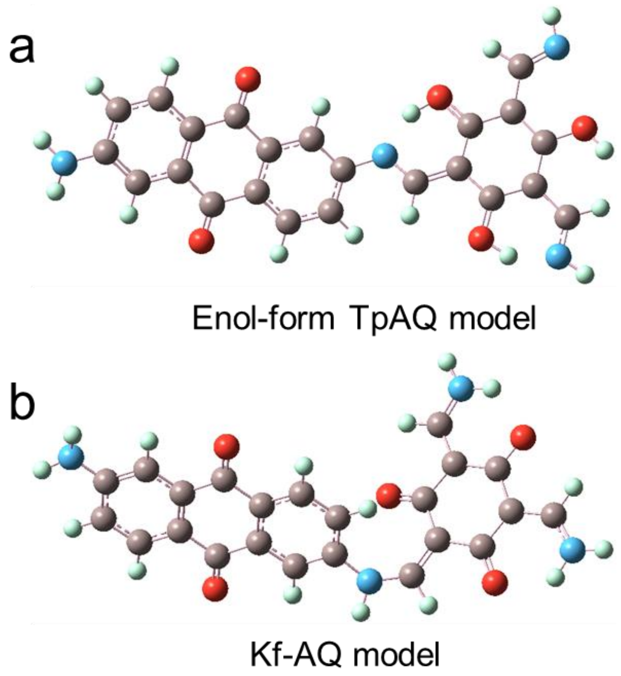

Figure S28. DFT calculation model. DFT calculation models of enol-form TpAQ (a) and Kf-AQ (b).

Supplementary Tables

Table S1. Fractional atomic coordinates. Fractional atomic coordinates for the unit cell of Kf-AQ.

**Kf-AQ (eclipsed AA stacking)**			
Space group: P6/m			
a = 30.59 Å, b = 30.59 Å, c = 3.51 Å			
α = 90.00, β = 90.00, γ = 120.00			
C	0.37164	0.67137	0.00259
C	0.35803	0.61843	0.00258
C	0.30756	0.58083	0.00258
C	0.26807	0.59421	0.00259
C	0.29526	0.53077	0.00257
C	0.33242	0.51808	0.00256
C	0.38262	0.55534	0.00255
C	0.39514	0.60527	0.00256
N	0.21828	0.72228	0.00263
N	0.42143	0.54331	0.00254
O	0.41533	0.70392	0.00259
O	0.22438	0.56176	0.00259

Table S2. Kinetic constants of k_f and k_d. The kinetic constants of k_f and k_d at different pH conditions.

pH value	k_f	k_d
7	13.12 ± 1.21	0.017 ± 0.001
9	12.12 ± 1.01	0.012 ± 0.005
11	20.91 ± 1.54	0.029 ± 0.002
13	31.39 ± 1.35	0.031 ± 0.002
14	26.34 ± 1.32	0.036 ± 0.005

Table S3. AQY calculation parameter. Details of the AQY calculation for Kf-AQ under various monochromatic light illumination (pH=13).

Wavelength (nm)	Light intensity (W/m^2)	H_2O_2 yield (mol)	Irradiation area (m^2)	Irradiation time (s)	AQY (%)
400	24	10.23×10^{-6}	3.74×10^{-3}	3600	15.8
450	30	9.27×10^{-6}	3.74×10^{-3}	3600	10.2
550	75	8.56×10^{-6}	3.74×10^{-3}	3600	2.50
600	95	6.96×10^{-6}	3.74×10^{-3}	3600	2.03
650	74	4.88×10^{-6}	3.74×10^{-3}	3600	1.51

Table S4. SCC calculation parameter. Details of the SCC calculation for Kf-AQ.

pH value	7	9	11	13	14
$\boldsymbol{n_{H2O2}}$ (mol)	4.3×10^{-4}	5.0×10^{-4}	6.1×10^{-4}	7.9×10^{-4}	7.5×10^{-4}
SCC (%)	0.38	0.44	0.53	0.70	0.66

Supplementary References.

1 Wu, Q. *et al.* A metal-free photocatalyst for highly efficient hydrogen peroxide photoproduction in real seawater. *Nat. Commun.* **12**, 483 (2021).

2 Shiraishi, Y. *et al.* Resorcinol-formaldehyde resins as metal-free semiconductor photocatalysts for solar-to-hydrogen peroxide energy conversion. *Nat. Mater.* **18**, 985-993 (2019).

3 Yang, C., Wan, S., Zhu, B., Yu, J. & Cao, S. Calcination-regulated microstructures of donor-acceptor polymers towards enhanced and stable photocatalytic H_2O_2 production in pure water. *Angew. Chem. Int. Ed.* **61**, e202208438 (2022).
